# Assessing the feasibility of establishing a centre of excellence in health logistics in the East African Community

**DOI:** 10.1186/2052-3211-7-S1-O16

**Published:** 2014-12-17

**Authors:** Philippe Jaillard, Lloyd Matowe

**Affiliations:** 1 Agence de Médecine Préventive (AMP), Ferney-Voltaire, France; 2 Pharmaceutical Systems Africa, Monrovia, Liberia

## Background

The East African Community (EAC) seeks to address challenges of vaccine and other commodities supply chain management by addressing weaknesses in human resource capacity. To achieve this EAC seeks to establish a Center of Excellence (CoE) with the objective of professionalizing health and immunization and related commodity logistics management in the region. The proposed framework for the CoE will be modelled according to the existing LOGIVAC reference center for health logistics for West African countries, in Benin. To advance this work AMP provided technical assistance to EAC to conduct a feasibility assessment.

## Method

The assessment methodology was adapted from the Human Resources for Supply Chain Assessment Guide and Tool developed by USAID|DELIVER in conjunction with PtD, and the Competency Compendium for Health Supply Chain Management developed by PtD. Specifically, the approach used desk reviews, consensus workshops, key informant interviews and analyses of existing systems.

## Results

In the East African Community, most SCM activities at the Central level are performed by pharmacists but non-pharmacists also play a significant role. At the facility level, SCM functions are mainly performed by nurses and midwives. In most countries EPI SCM functions are performed by public health technicians, clinical officers, nurses and midwifes. In all but one partner state there is at least one school of pharmacy. Even though pharmacists are being trained, current curricula for the pharmacy training does not adequately address SCM functions. In addition, curricula for nurses and midwives contains limited SCM. In all EAC Partner States, SC managers for EPI are mainly trained on the job.

## Discussion

There are SCM training gaps in the EAC with most of the countries having inadequate numbers of pharmacists. Also, pharmacy assistants/technicians are in short supply across countries. In addition to insufficient numbers of SCM cadres, no course currently exists to train a specialized SC cadre. Institutions that provide courses in SCM exist but lack sufficient capacity to produce quality courses. In addition to the shortage of trained personnel, there are also insufficient numbers of SC/logistics management academics. Curriculum strengthening and academic capacity building initiatives are required to adequately address SCM HR challenges.

## Lessons learned

A number of cadres, apart from pharmacists perform supply chain functions in the East African Community. Even though most EAC countries train pharmacists, these are in inadequate numbers and current curricula do not adequately prepare staff for SCM functions. Approaches to strengthen human recourse capacity for SCM are necessary to streamline SCM efficiency in the EAC.

